# Illinois State Dental Society

**Published:** 1886-07

**Authors:** 


### ILLINOIS STATE DENTAL SOCIETY.

REPORTED EXPRESSLY FOR THE INDEPENDENT PRACTITIONER.

Continued from page 318.

THURSDAY MORNING'S SESSION.

It was moved that at the next meeting the committee on Dental Art and Mechanism be instructed to secure a convenient room to be especially devoted to the exhibition of new appliances and materials, and that exhibitors shall be permitted to explain and demonstrate their inventions only in this room, between the sessions.

Dr. Black was called upon for his report, and said: You have all seen the results of these growths, in the formation of acids. I take the broth, which at the time of infection was neutral, and in a short time I find it acid, and the degree of acidity is in direct proportion to the growth of the vegetable organisms. And now arises a question in physiology. Where does this acid come from? It is the result of the life that gives us organic chemistry. The elements of the globe tend to the formation of permanent compounds. Without the intervention of some outside force there would be no change in nature. All would be at a stand-still. But the different forms of force are ever active; the sun, light, heat, winds, floods, etc., are constantly engaged in disrupting and intermingling the elements, and all tend to a disturbance of atoms and their remoleculization. These are some of the physical forces that assist in the changes of matter. The other force that is active in this work is life force, and this of itself is sufficient, assisted by such natural forces as are essential, to remodel all of organic nature.

It should be remembered that all Life is one, throughout the whole creation. Digestion in the vegetable world does not materially differ from that of animals. The lower forms of conscious existence have essentially the same general structure with man, and their life is one with ours. Food, pabulum, is taken into some form of a stomach, and it there meets with a digestive fluid which dissolves and fits it for assimilation, and it is finally woven into tissue, which becomes in time effete and is cast out as urea. Every form of life has within it the power of molecular changes and reorganization. The atoms are arranged and rearranged around a common center, first as tissue, and again as waste products. The *Torula*, or yeast plant, reorganizes pabulum, with the consequent formation of alcohol and carbonic acid. In the human digestive tract the fungi form peptones, and convert starches into glucose and levulose, and finally, by some mysterious process, produce lactic acid, as has been abundantly proved by Dr. Miller.

In the larger world we have poisonous plants, and those which possess medicinal properties, while others are inert. So with the fungi of the mouth; some produce one thing, some another. We do not know the physiology of the most of them. Although we have studied a part, there yet remains a great field for research. We know comparatively little of the conditions under which these fungi are formed. They seem to present widely different phenomena. In one mouth they grow and produce a great deal of acid, while in another their growth is very slow. Under certain circumstances these micro-organisms attenuate, or lose their virulence, as Pasteur has demonstrated. Perhaps, when we sufficiently comprehend their growth, we may be able to attenuate them out of existence. We find that some organisms grow freely in beef-broth, while others demand the presence of peptones. In some, the animal body is their natural habitat, while others perish when introduced into a living organism.

It is not necessary that I should narrate all the various media employed for their growth, but I may describe one of the most common. Take a pound of beef, place it in a vessel and bruise it thoroughly. It is then boiled for an hour or two, till the fluid, which has been added, clears up. This broth will usually be found acid, and though some forms will grow in it freely, for others it is essential to carefully neutralize it. This may be done with carbonate of soda. The sugar, or whatever is demanded by the organism, is added. If pepsine is necessary it is added, and time given it in which to work. To sterilize it you should boil it for an hour, when everything will be devitalized, except a few forms like *penicilium glaucum,* which are not destroyed by a boiling temperature. It is not necessary to exclude the air. It is exceedingly hard to eradicate the idea that air of itself causes decomposition, but it is not so. Air will not infect, if the germs which it contains are excluded. The culture tubes are prepared by first stopping them with cotton plugs, to filter out the floating germs. They are then placed in an oven and heated till the cotton is changed to a brown color, showing the beginning of the charring process, and this is my index of complete sterilization. The hot broth is now poured into the sterilized tubes, and they are, when cooled, ready for infection.

During the cooling, and in the handling, some of the tubes will become infected by stray germs which obtain entrance. To determine this the tubes are placed in the incubator for a time, to see if any germs spring up. When they are found free from intrusive organisms, they are duly infected. This infection is accomplished by dipping a platinum wire, which has previously been heated to redness, into the infectious mass, and carefully carrying a minute drop to the culture tube. I show you one that was thus infected yesterday. You will see that the growth is very weak and slow, the tubes showing but a very little cloudiness. Here is another in which the growth of caries fungi is very rank. This other tube, that I show you, was some of the same beef broth, duly sterilized, and which has remained in the incubator uninfected, as a control. The control tube, you will see, as I take from it a drop on the previously heated platinum wire and touch it to both red and blue litmus, is quite neutral, while this other infected tube, you see by its action, is strongly acid. The tube in which was the weak growth, you will observe by this other test, is but slightly acid.

Robertson, in 1835, said that caries was produced by fermentation, but he did not comprehend the principle of the process. We have all believed that caries was some kind of a chemical process. Dr. Watt declared this, but he thought the acids were produced by another process, and believed them to be inorganic in their nature. That much he affirmed through following Liebig. The germ theory is not a new one, but the knowledge of the manner in which chemical processes are brought about is modern. We had long been searching for the origin of the acids which are found in the mouth. We now know that when many of these mouth fungi are grown in the proper medium, they produce an acid which dissolves the lime salts. As the fungi would not proliferate in an acid medium, they would be self-limiting, were it not that the medium is again rendered neutral by the uniting of the acid with tooth bone, which allows the organisms to penetrate further, and again carry on their destructive work. They grow as vines would grow in a lattice work, the acid preceding them continually. Some species produce a gelatine, which acts as a protection. They crowd and dilate the dental tubules, and it was this appearance which Tomes saw, and pronounced a zone of resistance. Leber and Rottenstein carried on the work of observation, and made important additions to our knowledge. Milles and Underwood did excellent work, but they failed to study the physiology of the fungi. Miller did this, and he first triumphantly demonstrated the products of the organisms, and this, you will allow me to say, was the most perfect work of the kind that the world ever saw. His observations were so scientifically conducted, and the product so carefully analyzed, that he left not a hook upon which a tenable objection could be hung.

These acids are formed in the mouth and act upon the teeth while in their formative state, and we know that chemical combinations are most powerful in a nascent condition. If a man fails to eliminate the effete matter, he is poisoned by it, and this is true through all nature. So, if the acids of these organisms be not neutralized, they are suffocated in their own excrement, or product. Here is a tube in which I have neutralized the acid, and thus eliminated the waste products. This I continued to do until, as you see, the tube is almost full of gelatine. Had I not accidentally permitted the intrusion of another fungus, which destroyed the culture, I could have filled the tube. To neutralize the acid I made a lactate, using lime, and obtaining the lactate of lime, which is neutral and will not embarrass the fungus. When sugar was not added to this culture the fungus did not grow, for it did not find the proper nutriment. It must have sugar, or starch, which can be converted into sugar, for its nutriment. It will be observed, then, that even though all the fungi be not removed from a tooth cavity, if it be sealed up hermetically they will be starved to death, as grass would die from lack of nutriment and moisture. But the spores of these organisms will live for a very long time, even if deprived of nourishment, though they will not grow.

*Dr. Taylor*—What is the best germicide to use for the destruction of these organisms?

*Dr. Black*—I do not know. There are many things which I have not yet found out. It is not always caries fungi that form acids. We find many forms under artificial plates, and these produce acids, and that accounts for the way in which teeth that have clasps around them are sometimes girdled. It also explaines why mouths may become sore under plates that are not perfectly cleansed.

*Dr. Ames*—Does sterilization require a boiling heat?

*Dr. Black*—I would not like to risk anything less. I am in the habit of adding salt to raise the boiling point. Some fungi are destroyed at lower temperatures, but some are not killed at even the boiling point of water. Pathogenic organisms are, however, all destroyed by an hour's boiling. *Penicilium,* and a few other inert ones, will withstand that.

*Dr. Brophy*—Do you not differentiate between ferments of the stomach and the fungus ferments?

*Dr. Black*—Yes. There is a wide difference between the organic and inorganic ferments. The *Enzyme* convert starch into glucose and levulose, and then the process ceases. They will convert flesh into peptones, but they will not carry the conversion further. The ferments proper, the organic ferments, induce a growing which repeats itself. They produce a remolecularization into their own tissue, and this into waste products. This process repeats itself indefinitely, so that if the proper pabulum be provided, it is endless. Between organic fermentation and putrefaction no line can be drawn.

*Dr. Koch*—Suppose you were to plant together different forms of the cocci; will they hybridize ?

*Dr. Black*—I cannot tell, but I have often had different forms growing together, and they always have kept separate and distinct.

The Supervisor of Clinics, Dr. Frank H. Gardiner, of Chicago, made his report. A number of different operations in surgical, operative and mechanical dentistry had been made the day before, and the results were faithfully and fearlessly reported. Clinical exhibitions are not of as much benefit as they might be, because the failures and deficiencies in the operations are seldom pointed out, and hence young operators are led to accept as models for their future guidance methods and manipulations which really are not commendable. Operators at clinics usually work at a great disadvantage, and are obliged to resort to make-shifts to supply needed appliances, but even though great ingenuity may be displayed in accomplishing their work, the results should not be applauded or accepted as models unless they are perfect.

The report gave the names of the operators, but as some of them were severely criticised, they will not be repeated here.

Examinations of the clinical operations for the year before were made, and the condition reported. That this may be practicable, it is usual to select members of the society for patients. The operations of three of the clinical demonstrators of 1885 were found in good condition. One, performed according to the Herbst method, had proved a complete failure. There was considerable leakage at the cervical margin, and the filling was so poorly consolidated that it was half out. The operator subsequently explained that this was the result of his own inexperience, and not the fault of the system.

In the clinics of 1886, one operator filled the posterior masticating surface of the second bicuspid, using Perry's separators and

Brophy's matrix. The principal criticism was on the preparation of the cavity, which was declared to be improper.

Another operation of filling a posterior cavity by the aid of Dr. Call's matrix was pronounced a partial failure, owing to an insufficiency of space. Had more space been gained there would have been room enough to perform a better operation.

A gold crown was placed upon a second bicuspid. This was pronounced an imperfect operation, the palatine edge of the crown not reaching and covering the edge of the root, the occlusion being bad and the setting faulty. The tooth should have been filled instead of crowned. Another operation was that of filling a mesial cavity in a right superior cuspid, and the distal surface of the lateral incisor, the object being to demonstrate the practicability of filling teeth without previously gaining space. The operation was pronounced imperfect, because the teeth were cut away too much, and a permanent unsightly space left.

The mechanical clinics were reported as successful. The report recommended that operators should provide themselves with all the instruments necessary, that they might not be embarrassed through their absence, and thus be unable to do as good work as that of which they were capable. "Operators," said Dr. Gardiner, "should present good work, and not excuses."

### OPE[illegible]TIVE DENTISTRY.

This had been assigned as one of the special subjects for consideration at this session. A paper was read by Dr. C. R. Taylor, upon "The Preparation of Pulp Canals and Cavities for Filling."

*Dr. Davis*—No vocation demands a greater degree of honesty than does ours, for in no calling is it more easy to deceive those who demand services. A canal can readily be stuffed in an imperfect manner, and it may be some time before the poor work will be discovered. If it were the habit of all dentists to make the fee dependent upon the time spent, there would not be the temptation to hurry an operation at the expense of its thoroughness. To properly fill or treat a pulp-canal we must gain a perfect entrance that shall be in a line with the axis of the tooth. Enlargement of the canal is not necessary for treatment, and if liquid gutta-percha be used it is not essential for the filling. The rubber dam should be applied in all operations upon the pulp-chamber and canals of teeth, for obvious reasons. A great variety of instruments is not demanded, and the engine should be used as sparingly as possible. When it is employed, the burs used, as well as all other instruments, should be sharp and in perfect order. The operator should be fearless in cutting any overhanging dentine, and thoroughly painstaking in his cleansing.

*Dr. Hanaford*—I have been struck with the absence in our late literature of anything upon the capping of pulps. It is not long since this occupied attention, almost to the exclusion of other things. We now seem to have gone to the other extreme, and to be progressing backward. In a report published in *The Southern Dental Journal,* I saw something like this: "In the near future we shall find that the capping of pulps has been relegated to those who do not attend society meetings. The effects of this practice will be found in discharging abscesses, in blackened teeth, and in filthy mouths." I do not agree with this, but I believe that the capping of pulps that are unfit for it is but too common. It is better to extirpate at once, than to leave the pulp to die by stages.

For filling roots, plastic materials are now almost universally employed, and although there may be some inconvenience in using them in teeth with open foramens, there is little doubt that they are better than metallic fillings.

Crown work is becoming more popular each year. Too much has been said of the construction of crowns, and not enough about the preparation of the root. It requires shaping to a definite form, and corundum wheels and files are not sufficient for this work, as the roots are conical. Unless this form is materially changed the band is not in contact with the tooth at its distal edge, and it acts as a continual irritant. The root should be of the proper shape and the band then carefully fitted. It should be tried on, and the interior then wiped out carefully. If now there be a leakage, the band should be removed and made smaller.

We hear nothing now about permanent separation of the teeth. That is another topic which once absorbed great attention, but which is now fallen into desuetude. It is another instance of one extreme following another. Flat fillings, with a permanent separation, are usually protective of the tooth. If contour operations are to be made, there should first be secured abundant separation, or the work will be short lived.

*Dr. K. B. Davis*—I object to the making of retaining pits for starting a filling in bicuspids if there be a living pulp. There is no necessity for it. If the filling be commenced with soft foil, it will obviate the necessity for retaining pits. I object to the use of the mallet in beginning operations. I believe that many frail margins are in this way permanently injured. Soft foil should be employed until the cavity is half or two-thirds full.

*Dr. McKellops*—What holds the pieces together while you are inserting the filling, if there be no retaining points and soft foil is used ?

*Dr. Davis*—They are wedged together, and sometimes held by contact with the next tooth.

I object to the extraction of second bicuspids for regulating purposes. The instances are rare in which it is necessary to sacrifice a tooth.

Many fillings fail because of the neglect properly to finish at the cervical margin. I have seen gold fillings that projected a line at the magin of the gums, where was formed the nucleus of decay. Yesterday I had a tooth filled for the first time in fifteen years. It would be better for us if we could occasionally have a tooth filled, that we might remain in an active state of sympathy with our patients.

*Dr. Sitherwood*—Nearly every dentist fills roots of teeth with plastic materials. I have frequently forced gutta-percha into teeth that were abscessed until it came out of the fistulous opening. When there is any caries of the bone attending dead roots, it is very difficult to keep the canals dry. In such cases I would fill as quickly as possible, and treat the caries afterward. For periostitis succeeding the filling of root canals, there is nothing so effectual as Dr. Darby's Capsicum Plasters.

*Dr. Ottofy*—In the preparation of cavities for filling, the final examination should always be under a magnifying glass, to detect minute imperfections. I seldom cap an exposed pulp for a person more than thirty years of age. Good results cannot be confidently anticipated except in young people. If there shall have been much pain preceding the excavation, I seldom attempt capping.

*Dr. Solomon*—Believes in contour work, and by means of diagrams on the board he illustrated the principles involved. Flat fillings are uncomfortable in use, while the usefulness of a tooth is lost in proportion to the amount of its masticating surface that is removed.

*Dr. McKellops*—Asked about teeth that were deprived of periosteum. He referred to the alleged practice of Dr. Younger, who bores holes in the jaw and inserts teeth in them. He also referred to a case in which Dr. Morrison had extracted a tooth from one locality in the jaw and inserted it in an artificial cavity in another place.

*Dr. Barrett*—Said that if any miracles were to be performed he should look for their accomplishment in St. Louis, and the man there who could reverse the processes of nature, if anyone could, would be Dr. Morrison. He had heard of the case referred to by Dr. McKellops, and was assured by credible dentists that what most people would think but an apochryphal story, was a veritable fact. He could not conceive of the possibility of the existence of bone without its periosteum, nor of tooth without its pericementum. Nor could he comprehend how pericementum would proliferate in a gimlet-hole. But Dr. McKellops had thrown a new light upon the Morrison case by admitting that the tooth was inserted in what was a previous tooth-socket.

He detailed his method of obtaining an impression of a root of a tooth by using rolled copper wrapped about the end of the root, a cast being then taken with plaster of paris which filled the copper ferrule; then by wrapping more copper about the removed end and pouring melted tin into it, a perfect model of the end of the root was obtained.

In the filling of the roots of teeth there is a principle involved. There is but one tissue that needs protection, and that is the dentine. The cementum is otherwise nourished, while the enamel practically needs no nutrition. If a septic canal be made aseptic, and any opportunity for the entrances of septic organism be effectually stopped, it must remain aseptic. But there may be an exudation into an open pulp chamber, and hence it is safest to stop this entirely. For this purpose we need a plastic material, that it may fill all the inequalities of the pulp canal; a non-conductor that thermal changes may be avoided; a bland material that it may be as innocuous as possible. We do not wish a filling that has any medicinal qualities whatever, because we are now supposed to be through with medicaments, and the cavity to be entirely aseptic. It must be a simple material, and not a chemical compound, as the latter would be more likely to disintegrate. These qualities are only found in the hydro-carbon gums, notably gutta-percha, and hence that was the ideal filling for roots of teeth.

*Dr. Matteson*—To shape the roots of teeth preparatory to crowning, I use corundum disks of different sizes.

*Dr. McKellops*—That is easier said than done. In putting on crowns, I fit the band while the patient is in the chair, and dress the roots with a file that cuts towards the crown. For a gold crown I make the band of less width than the length of the crown demands. The crown part of the tooth is then made so that the band will telescope within it. They are then placed in the mouth and the bite adjusted, when they are removed and soldered together. For separating teeth I use waxed tape. I take plenty of time, and do not fill until the soreness of separation is gone. I have seen the whole socket and septums surrounding a tooth slough away because of too rapid wedging. I employ any device that I think will assist me, but am not wedded to any particular method. For impacting fillings, I use a Bonwill mallet, and contend that there is nothing so effectual. But I have modified the instrument.

Further discussion was, by vote, postponed till another session.

*Dr. Moody*—Offered a series of resolutions commending the course of Dr. Harlan as a member of the Board of Dental Examiners, and requesting the Governor to re-appoint him.

(TO BE CONTINUED.)

